# A non-contact interactive system for multimodal surgical robots based on LeapMotion and visual tags

**DOI:** 10.3389/fnins.2023.1287053

**Published:** 2023-10-17

**Authors:** Xinkang Zhang, Jie Wang, Xiaokun Dai, Shu Shen, Xinrong Chen

**Affiliations:** ^1^Academy for Engineering and Technology, Fudan University, Shanghai, China; ^2^Shanghai Key Laboratory of Medical Image Computing and Computer Assisted Intervention, Shanghai, China; ^3^Jiangsu High Technology Research Key Laboratory for Wireless Sensor Networks, Nanjing University of Posts and Telecommunications, Nanjing, China

**Keywords:** surgical robot, human-computer interaction, LeapMotion, Aruco, non-contact

## Abstract

In recent years, the integration of robots in minimally invasive surgery has gained significant traction in clinical practice. However, conventional contact-based human-computer interaction poses the risk of bacterial infection, significantly limiting the role of robots in surgery. To address this limitation, we propose an innovative interaction method rooted in gestures and visual tags, allowing surgeons to control and fine-tune surgical robots without physical contact with the environment. By encoding the six gestures collected using LeapMotion, we can effectively control the surgical robot in a non-contact manner. Moreover, utilizing Aruco technology, we have accurately identified the 3D spatial position of the visual label, and developed 12 fine-tuning operations to refine surgical instruments. To evaluate the applicability of our proposed system in surgery, we designed a relevant experimental setup. In the experiment, we achieved enough precision. These results demonstrate that our system meets the clinical standard, providing doctors with a non-contact and flexible means of interacting with robots during surgery.

## 1. Introduction

With the rapid advancement of sensor technology and computer technology in recent years, the capabilities of robots and manipulators have become more sophisticated and performed better. As a result, they have become increasingly prominent in various aspects of daily life and specific fields. This surge in demand for human-computer interaction and the development of human-computer interaction technology have led to a proliferation of mainstream methods, including joystick buttons, voice interaction, and gesture recognition, among others. In recent years, human-computer interaction has emerged as a popular research area in the fields of smart homes, customer service, remote control, and medicine.

In the medical field, human-computer interaction technology is widely utilized for rehabilitation purposes, assistance for those with disabilities, and surgical robots (Díaz et al., 2014; Ohmura et al., 2018; Nagyné Elek and Haidegger, 2019; Cao et al., 2022a). Chen C. et al. (2023) decompounds muscle EMG signals to quantify neural features and map them to three-degree-of-freedom wrist movements through a multiple linear regression model. This method has shown great potential in the reconstruction process. Chen W. et al. (2023) employs myoelectric signals from the lower limbs to control exoskeletons. The quadriceps and hamstring muscles are selected to obtain gait information, and 16 dry electrodes measure electromyography signals transmitting information to a host computer via Bluetooth. The processed signals help users control their gait effectively. Ali et al. (2023) proposes a deep learning-based Thought-to-Text conversion for patients with neurodegenerative diseases like Alzheimer’s disease type through EEG. Collected EEG signals are preprocessed with a band-pass filter and divided into five classifier tasks using XGBoost (Ogunleye and Wang, 2019) classifier. Finally, the CNN-LSTM deep neural network (Mutegeki and Han, 2020) learns advanced features from MI-EEG signals and translates them into corresponding alphabets. Coughlan et al. (2020) utilizes machine vision to provide voice prompts for visually impaired patients. Specifically, the Camera Input–Output (CamIO) augmented reality tool guides the patient in 3D space using a pen covered with 3D visual labels, leading the patient closer to the target.

In the realm of surgical robots, various information modalities will also be utilized to assist medical professionals (De Rossi et al., 2021; Long et al., 2021; Cao et al., 2022b). Surgical robots possess distinct advantages over humans, including unparalleled precision, exceptional stability, and rapid execution. As a result, they have become increasingly prominent in endoscopic surgery (Kang et al., 2009; Gifari et al., 2019) in recent years.

According to Jacob et al. (2011), a system is proposed to track the surgeon’s hands, identify required surgical instruments, and have the robotic arm pass them to the doctor. However, this system is still slower than manual work, and nurses who pass instruments themselves do not need to leave the operating table, indicating a weak irreplaceability in surgery. Next, referring to Van Amsterdam et al. (2022), a video-based surgical assessment system is proposed, including automatic activity recognition, technical skill assessment, book assistance, and other functions. By integrating a multi-modal attention mechanism into a dual-stream temporal convolutional network, real-time dynamic weight kinematics and visual representation calculation improve fine-grained surgical data analysis accuracy. However, these methods have a common problem: complexity and single modality interaction. To address this, different interaction methods need to be introduced. Moving on, Cho et al. (2018) aims to establish a non-contact computer-assisted surgery system. LeapMotion’s mature gesture recognition module is used to obtain hand gestures, and features are manually extracted before being aligned with support vector machine (SVM) classification (Chang and Lin, 2011). This creates a non-contact control interface with gesture recognition functionality. Similarly, Dwivedi et al. (2019) combines myoelectricity and visual tags to manipulate a robotic arm. Kam et al. (2018) provides three-dimensional attitude information to control the robotic arm’s position, while processed myoelectric signals control the bionic hand at the arm’s end for grasping actions. In our previous work (Wang et al., 2023), we explored gestural interactions with various countries to achieve seamless human-computer interaction and contactless operation. However, a common limitation is that users must maintain a predetermined posture to perform a single trigger on a specific action, which lacks the dynamic flexibility of more complex gestures.

Most of the existing systems rely on complex technology and have high equipment requirements. Therefore, finding a balance between simplicity and robustness is crucial. In this paper, we propose a straightforward method that integrates consumer-grade gesture recognition technology LeapMotion with accurate 3D space perception technology based on visual labels Aruco. This framework allows surgeons to interact with surgical robots without physical contact, leveraging the potential of surgical navigation systems. By doing so, we can maximize the capabilities of surgical robots in surgical procedures.

## 2. Materials and methods

### 2.1. System composition

**Figure 1:**
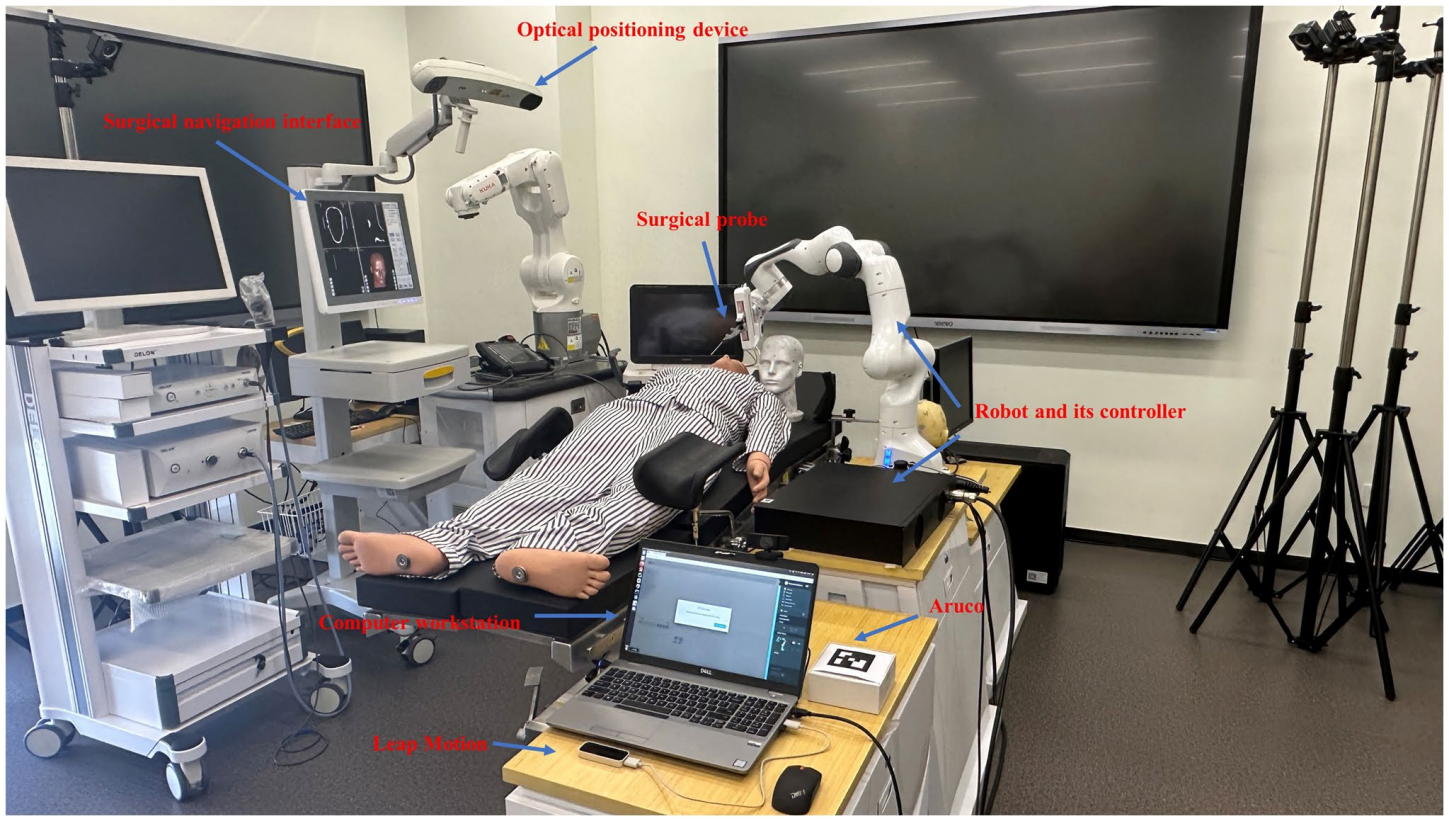
Overview of the surgical navigation robot system.

The navigation operating system is primarily composed of a computer workstation, a gesture recognition module, an Aruco recognition module, a surgical navigator based on optical positioning, a surgical probe, a positioner, and a robot module comprising a seven-axis robotic arm and a control cabinet. All navigators and operating tables can be moved according to the position of the patient and doctor. One of the workstations is connected to the surgical navigation system and robotic system. The surgical navigation system tracks the precise position of surgical instruments and patients in real-time using the locator installed on the surgical instrument and the operating table, which consists of an array of reflective balls. Doctors can operate the collaborative surgical robot and its end-effectors with great flexibility through gesture recognition and tag-based recognition technologies. The entire surgical navigation system creates an enhanced surgical space, aligning the target area or target point in the preoperative medical image with the real patient’s body to provide visual assistance for the doctor. In such cases, gesture and visual tag information can be used as a remote, aseptic method to adjust the position of surgical instruments (Figure 1).

### 2.2. System workflow

**Figure 2:**
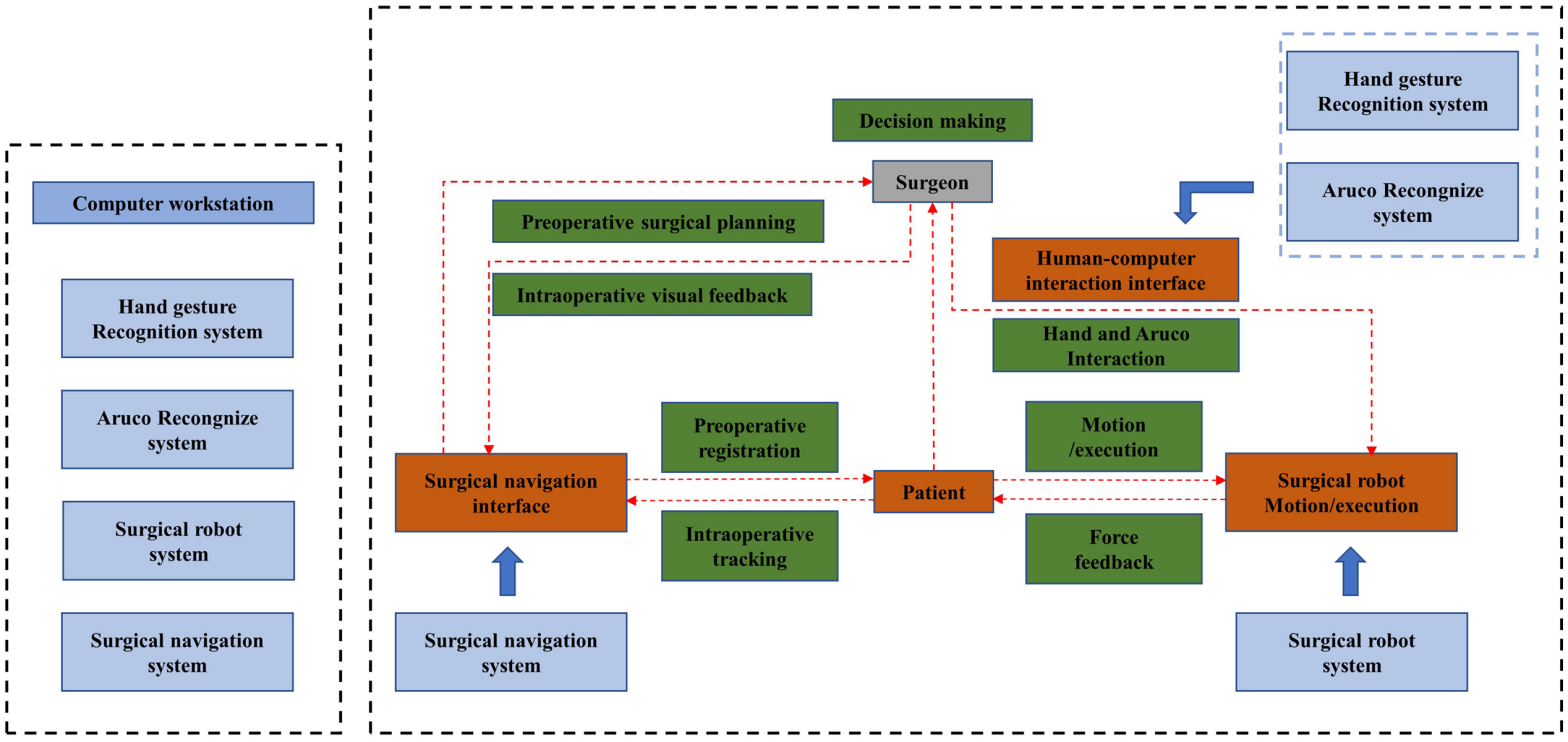
Workflow of the surgeon-robot system.

The proposed whole surgical system is depicted in Figure 2. The computer workstation acts as the central control unit to regulate the robot, generate enhanced surgical imaging data, and facilitate human-computer interaction. The hardware component of the collaborative interactive surgical robot system is represented by blue in the figure. In addition to the operator, surgical navigation interface, surgical robot movement/execution, patient, and contactless interaction system, the figure also depicts the data flow of hardware-participant interaction, indicated by black arrows, and the surgeon-centric flow of interaction, shown using red arrows. To achieve varying degrees of operational flexibility under non-contact conditions, a system employing non-contact human-computer interaction is proposed. The surgeon must adjust the position of the surgical instrument or operate the surgical instrument according to a predetermined trajectory during surgery. The surgeon can move and fine-tune the surgical robot through contactless gesture control and a special marker with visual markers. The article’s core is to utilize two types of sensors and decoding technologies to enable seamless and flexible interaction between the surgeon and instrument during the procedure using non-contact means.

### 2.3. Gesture interaction based on LeapMotion

**Figure 3:**
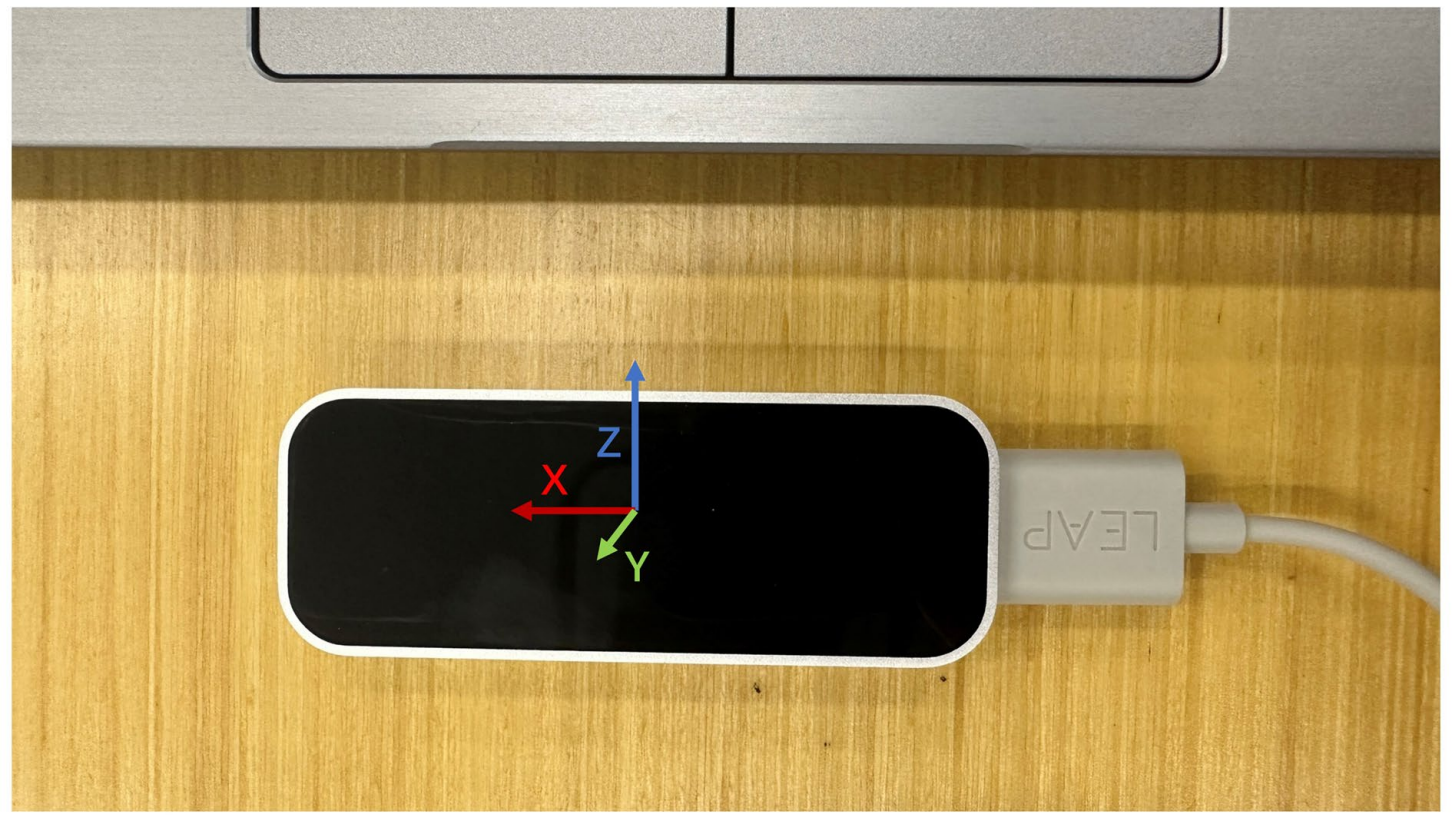
The LeapMotion used in this work.

In this section, our attention is directed toward the gesture recognition module based on LeapMotion. The specific architecture of the model is illustrated in Figure 3.

Gesture information collected via LeapMotion is utilized to rapidly adjust the position of the surgical instrument held by the robotic arm. LeapMotion is a device used to collect gestures, and the collected information includes the overall position of the hand, the position of key points on the hand, the length and direction of the fingers, and other related data. In this work, LeapMotion will continuously obtain timing frames, and the pose data will be parsed and expressed in a form that is convenient for us. The posture data will then be decoded into instructions and transmitted to the surgical robot module to perform the corresponding operation. For the simplicity and robustness of the instructions, we have defined six instructions to control the robotic arm to move quickly and over a large range.

#### 2.3.1. Introduction and principle of LeapMotion equipment

LeapMotion is a somatosensory device developed by LEAP, which specializes in recognizing the geometric information of the hand. The device consists of two cameras and three infrared LED lights, allowing it to obtain images from two angles and depth information using infrared light. The binocular camera’s principle is based on the human visual system, making LeapMotion more accurate and reliable in hand recognition. Its recognition accuracy reaches one hundredth of a millimeter, which is more accurate than Microsoft’s Kinect and has more advantages in gesture interaction. Additionally, LeapMotion has a lower price compared to data gloves, making it a more cost-effective option in most scenarios, with sufficient precision (Table 1).

**Table 1:** Accuracy of hand recognition with predefined categories.

Category	1	2	3	4	5	6
Accuracy	95%	97%	99%	96%	98%	98%

#### 2.3.2. Reading and data processing of LeapMotion

LeapMotion’s two cameras capture images from different angles and reconstruct the 3D information of the palm in space. The detection range is approximately 2.5–60 cm above the sensor. The coordinates of the entire space are as follows: the Cartesian coordinate system is centered on the sensor, and the *X*-axis of the coordinate’s points to the right, the *Y*-axis points upward, and the *Z*-axis points away from the screen. The unit of the output distance value is millimeters. Each frame of information contains the position and orientation of the center of the palm, as well as the position and pose information of each key point on the hands and fingers. The approximate structure of the read structure is shown in Figure 3.

To represent manipulation commands, certain hand features must be extracted. In the interaction design of this article, specific keypoint distance and normal features are selected as features to reflect the uniqueness of different hand poses. In the interaction design, there are two overall postures of the hand: one where the palm is facing down, and the other where the palm is facing inward. The orientation can be determined by the normal vector of the hand. To determine the orientation, the method used is to calculate the cosine similarity between the vector and the standard coordinate axis vector. The similarity ranges from −1 to 1, and the greater the similarity, the closer the two vectors are aligned. From this, we can obtain the palm direction information.

To detect finger poses, distance features between key points are extracted, specifically, whether the fingers are bent or stretched based on the distance from the fingertip to the base of the palm. And determine the command according to the gesture of the finger.

The hand information in each frame can be extracted from the frames read out from the device, including the first 3D coordinate $x_{start-i}\in R^{3}$ and the tail 3D coordinate $x_{end-i}\in R^{3}$ of each finger. In addition, the position of the root $x_{root}\in R^{3}$ of the hand can also be obtained.


(1) $$s_{i}=\left\{\begin{array}{c} 1\;\mathrm{if}\;\;d\left(x_{start-i},\;\;x_{root-i}\right)\geq threshold\_d\\ 0\;\mathrm{if}\;\;d\left(x_{start-i},\;\;x_{root-i}\right)<threshold\_d \end{array}\right.$$

where $s_{i}$ indicates the state of the *i*-th finger, and threshold_d represents the threshold for judging the state of the finger, d is an operator used to calculate the Euclidean distance.

To take palm orientation into account, two vectors are used to represent.


(2) $$d_{L}\in R^{3}$$

(3) $$d_{R}\in R^{3}$$

where $d_{L}$ indicates the palm facing direction of the left hand, while $d_{L}$ indicates the palm facing direction of the right hand.


(4) $$S_{L}=\left(s_{0},s_{1},s_{2},s_{3},d_{L}\right)$$

(5) $$S_{R}=\left(s_{0},s_{1},s_{2},s_{3},d_{R}\right)$$

where $S_{L}$ indicates the state of the left hand, while $S_{R}$ indicates the state of the right hand.

The state of the two hands will be decoded into different operating instructions, corresponding to different actions.

### 2.4. Interaction based on Aruco

A second-stage fine-tuning of the manipulator pose and position is carried out using an Aruco-based pose estimation method. Aruco is a widely used label in computer vision localization tasks and augmented reality applications, first proposed in the paper (Garrido-Jurado et al., 2014) in 2014. By placing Aruco on the object to be estimated or tracked, attitude estimation and tracking of the object can be achieved through its posture. In this paper, Aruco is used as a means of manipulation, and the operation action is obtained by detecting its posture. Through rotation and displacement in different directions, a total of 12 operating instructions, corresponding to 12 distinct actions, are generated. These instructions are used for fine-tuning the robotic arm in a limited range.

#### 2.4.1. Encoding

The full name of the Aruco code is the Augmented Reality University of Cordoba, which is visually represented as a square with a black background, and the grid pattern inside the square serves as a distinct identifier. The size of the detected square is a crucial reference information for estimating 3D pose from monocular RGB images. In image editing tasks, the position information provided by the Aruco tag enables accurate processing of the perspective relationship of the image. In scenarios where robots are required to be positioned, such as automated warehouses, the Aruco mark can serve as both a positioning mark for robots and an identification mark for designated areas.

#### 2.4.2. Aruco generation

**Figure 4:**
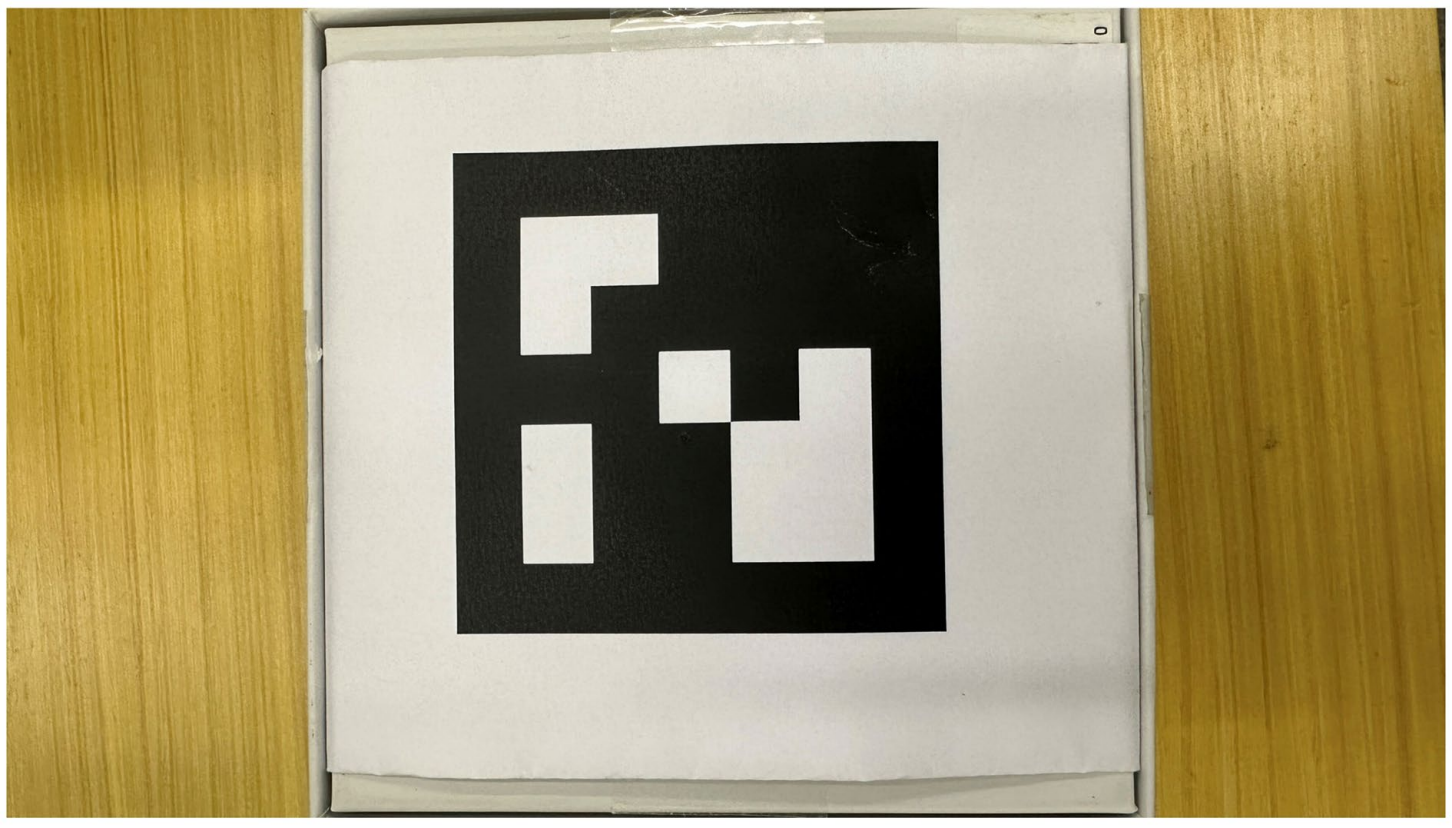
Aruco mark.

We use opencv to generate Aruco’s markers. There are 25 predefined markup dictionaries. All Arucos in each dictionary contain the same number of blocks. According to different parameters, such as size, id, border width, etc., Aruco codes with predefined patterns and sizes are generated as needed for manipulation. As shown in Figure 4, in this work, we employ Aruco codes of size 10 × 10.

#### 2.4.3. Pose estimation and parameter resolve

To detect the location and pose of tags, several steps are involved. First, the most prominent contour must be extracted using a local adaptive threshold method, which is highly robust to various lighting conditions. Next, contour extraction is performed, followed by a four-vertex polygon approximation. The resulting four-vertex polygon area is then passed through the homography matrix to eliminate perspective projection. Otsu’s binarization method is used for thresholding. The interior of the polygon is then divided into a grid to assign 0 or 1 individually. Finally, the result is matched with the result in the dictionary to obtain the id of the tag. The pose relative to the camera is estimated by minimizing the reprojection error of the corners.

Here, we use python based programming language and opencv to obtain the position and pose of labels in detail. Opencv is a widely used open source library for computer vision and image processing. It contains a large number of image processing tools and can help developers create applications such as machine learning, target detection and tracking, and image processing. Opencv is heavily used in both academia and industry. In order to help users develop quickly and concisely, aruco’s related interfaces have been integrated into the cv2.aruco library of opencv. We call several of the APIs to implement the label positioning function. Three APIs are mainly used: cv2.aruco.Dictionary_get() is used to obtain the dictionary of labels for corresponding label searches; cv2.aruco.detectMarkers() is used to detect labels from the image; and cv2.aruco.estimatePoseSingleMarkers() is used To estimate the pose of the label detected in the previous step and obtain the rotation and translation information.

We use these APIs to get the pose $R_{vec}\in {\mathbb{R}}^{3}$ and position $T_{vec}\in {\mathbb{R}}^{3}$ of Aruco. $R_{vec}$ represents rotation in three directions, which are $Ang_{pitch}$ , $Ang_{roll}$ , and $Ang_{yaw}$ . $T_{vec}$ represents displacement in three axes, which are $t_{x}$ , $t_{y}$ , and $t_{y}$ .


(6) $$s_{x}=\left\{\begin{array}{c} 1\;\mathrm{if}\;\;t_{x}\geq t\_threshold\_pos\\ 0\;\;\;\mathrm{otherwise}\\ -1\;\mathrm{if}\;\;t_{x}\leq t\_threshold\_neg \end{array}\right.$$

(7) $$s_{pitch}=\left\{\begin{array}{c} 1\;\mathrm{if}\;\;Ang_{pitch}\geq A\_threshold\_pos\\ 0\;\mathrm{otherwise}\\ -1\;\mathrm{if}\;\;Ang_{pitch}\leq A\_threshold\_neg \end{array}\right.$$

where t_threshold_pos indicates the positive threshold, and t_threshold_neg represents the negative threshold. $s_{x}$ indicates whether there is a significant displacement on the *x*-axis. And A_threshold_pos indicates the positive threshold, and A_threshold_neg represents the negative threshold. $s_{picth}$ indicates whether there is a significant deflection in the pitch angle.


(8) $$S=\left(s_{x},s_{y},s_{z},s_{pitch},s_{roll},s_{yaw}\right)$$

where S indicates the status byte from Aruco. Among the six flag bits included in S, $s_{x}$ is calculated as shown in the formula above. According to the translation of the tag in the *x* direction in the three-dimensional coordinate system, when the translation is greater than the positive direction threshold, this direction flag is set to 1. When the translation is less than the negative direction threshold, set the direction flag to −1. If neither of the above two conditions is met, set it to 0. In the same way, the calculation method of $s_{y}$ is: compare the translation of the label in the y direction with the corresponding threshold, and obtain the flag position $s_{y}$ in the *y* direction; the calculation method of $s_{z}$ is: compare the displacement of the label in the *z* direction with the corresponding threshold. Compare and get the flag bit $s_{z}$ in the *z* direction. Similarly, $s_{pitch}$ is a flag bit obtained by comparing the rotation angle on the pitch axis with a preset threshold. Based on this, we can determine whether the label has a sufficient rotation angle in this direction. $s_{roll}$ is a flag for rotation on the roll axis, indicating whether the label has sufficient rotation in this direction. $s_{yaw}\;$ is a flag for rotation on the yaw axis, indicating whether the label has sufficient rotation in this direction.

### 2.5. Interaction with surgical robot

In stage 1, when quickly adjusting the position, use gestures to adjust. For example, if you want to control the movement of the robotic arm, make a corresponding action above the Leap Motion. After detecting that the gesture and direction of the two hands meet the corresponding conditions, start counting. When the threshold is reached, set the preset action flag to True. Start to transmit signals to the surgical robot. When the robot reaches the designated position, change the gesture to clear the marked position.

#### 2.5.1. Pose estimation and parameter resolve

In the second stage, the position of the Aruco tag is adjusted by translation, and its angle is adjusted by rotation, thereby triggering corresponding actions. Through the same mechanism, the instruction of the corresponding action is triggered, and the flag is set to true.

#### 2.5.2. Aruco attitude command calculation

**Figure 5:**
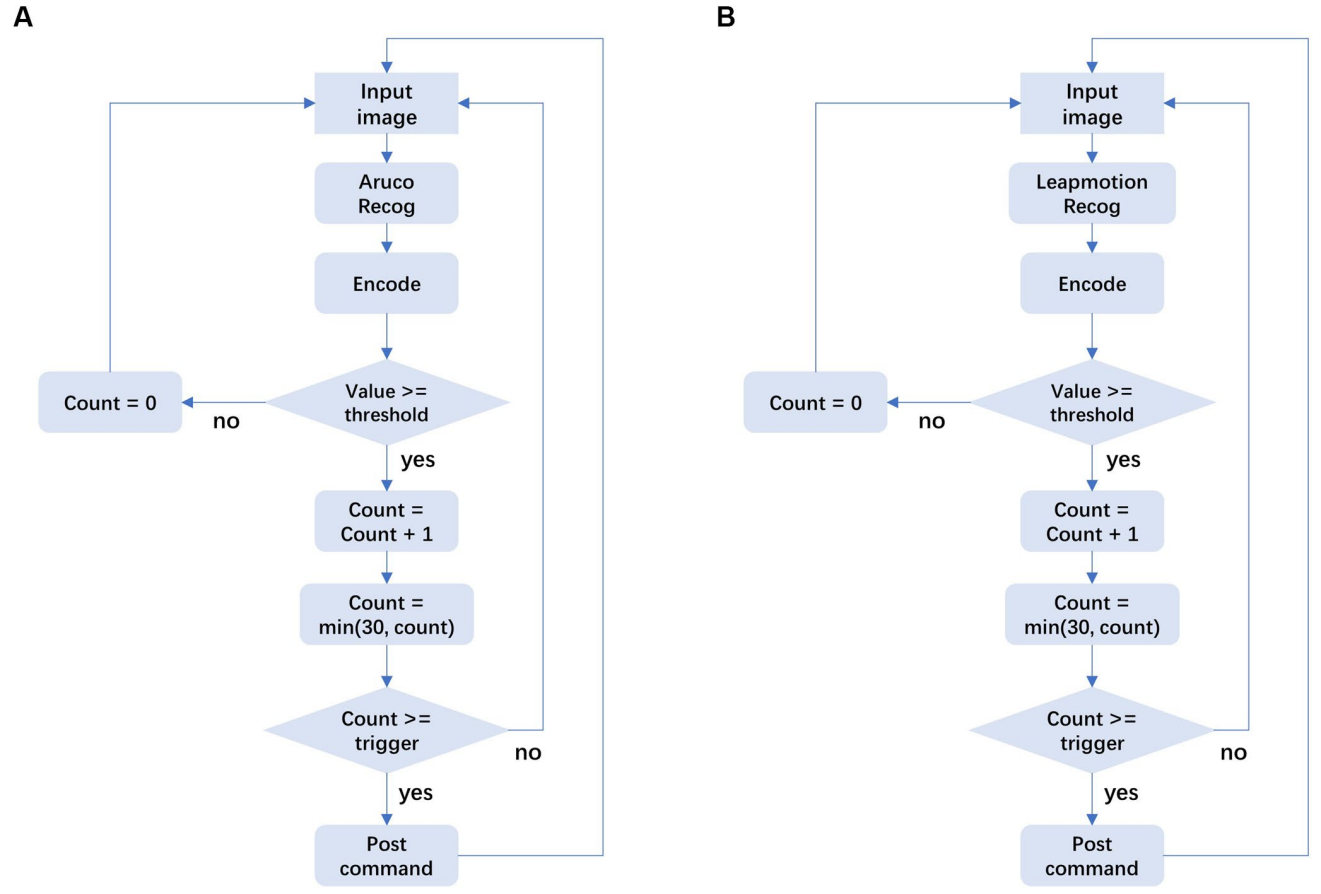
State machine design for HCI tasks.

After obtaining the position and attitude of the tag, we need to generate the corresponding operation instructions. In this part, we use a state machine commonly used in control systems to control the triggering of operation instructions. As shown in the flow chart on the left side of Figure 5, we set a counter. When the status of the tag meets the trigger condition of a certain instruction, the corresponding counter value will increase. When the trigger condition is met for more than 30 consecutive frames, we will send the corresponding operation instructions. In order to prevent false triggering, our program stipulates that if a frame does not meet the conditions, the corresponding counter will be set to 0, thus ensuring the stability of the instruction triggering process.

#### 2.5.3. Pose mapping to robot

**Figure 6:**
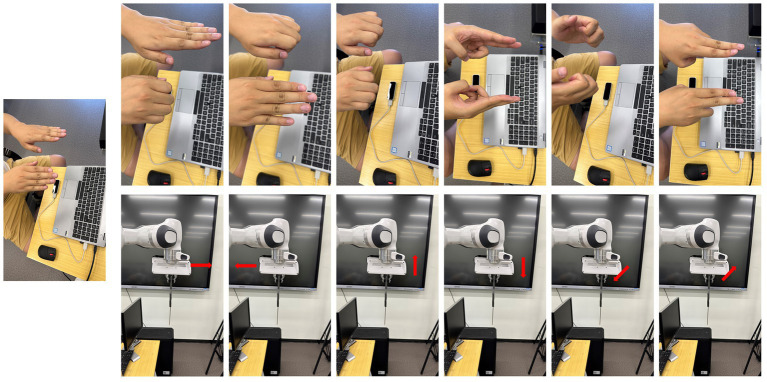
The hand gesture and corresponding action.

**Figure 7:**
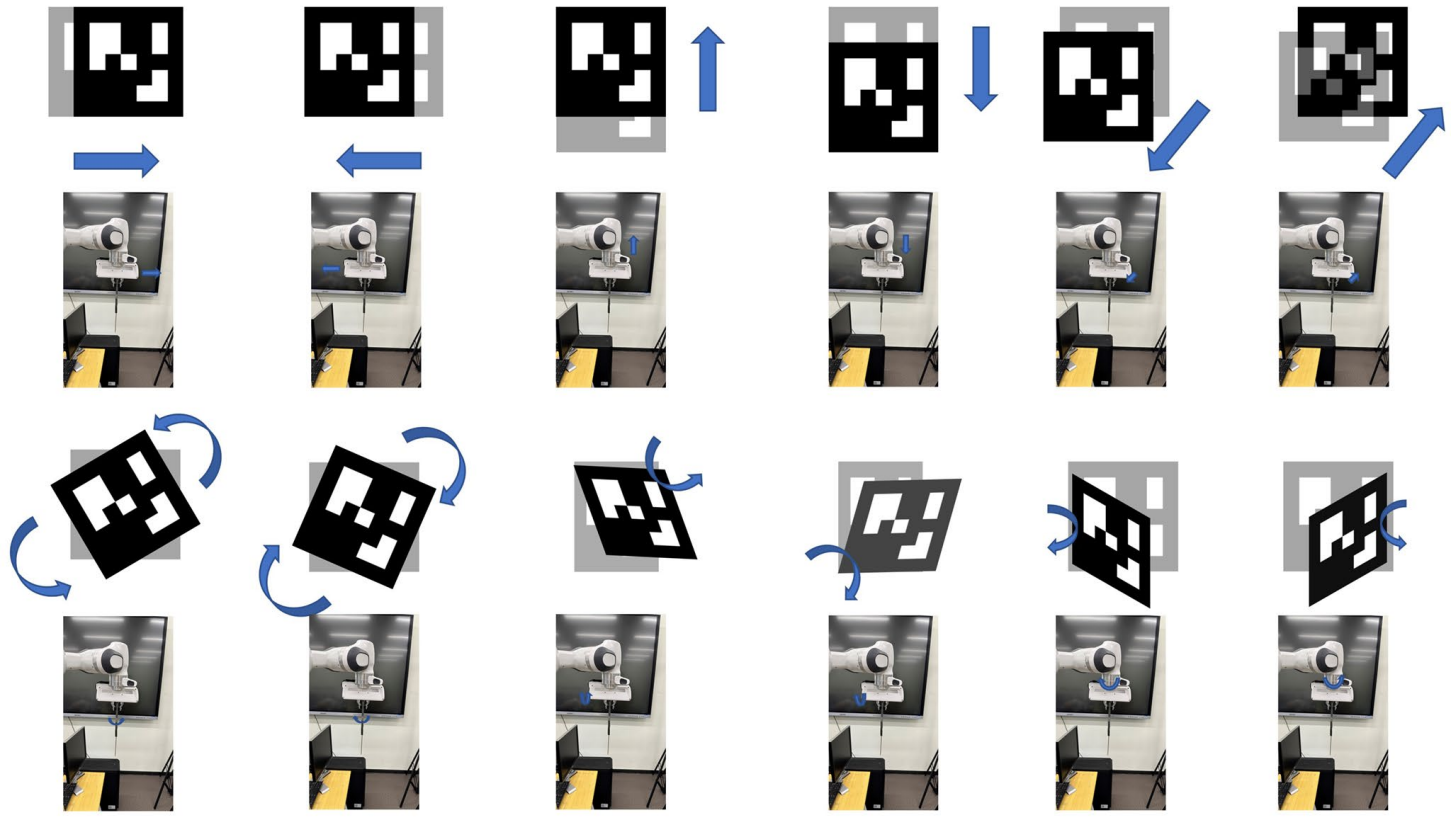
The Aruco gesture and corresponding action.

After obtaining the operator’s posture, it needs to be mapped to the robot end effector, and the corresponding inverse kinematics is calculated to obtain the joint angle of the target robot, and then the relevant operations are completed according to the operator’s control intention. In the design of this paper, we divide it into two mapping relationships according to the speed of the operator’s posture change. When the robot is far away from its operating object, the operator needs to control the robot to perform large movements, and the operator’s posture changes relatively quickly. Accordingly, we designed the first mapping relationship of fast motion, as shown in Figure 6, including forward, backward, up, down, left, and right based on the end effector, and the speed is 0.5 m/s. This mapping relationship ensures the moving speed of the manipulator under absolute safety and can reach the vicinity of its operating object as soon as possible. When the robot reaches the vicinity of its operating object, it needs to adjust the posture of the end effector according to the operating task, and reach the target point at a relatively slow speed for related replacement. To this end, we designed a second fine-tuning mapping relationship as shown in Figure 7, including forward and backward, up, down, left, and right, and end-effector as a reference with a speed of 0.1 m/s. Fine-tuning of the direction of the actuator as a reference, including left pick, right pick, up pick, down pick, clockwise rotation, and counterclockwise rotation. The trajectory movement and attitude adjustment of the entire desired are based on the Franka open-source library and the ROS control package. In order to realize the robot’s smooth response and real-time attitude tracking, a speed closed-loop controller suitable for the Franka robot is designed, and the control frequency is 1,000 Hz, which corresponds to the real-time communication frequency allowed by the Franka mechanical alarm system.

## 3. Results

**Figure 8:**
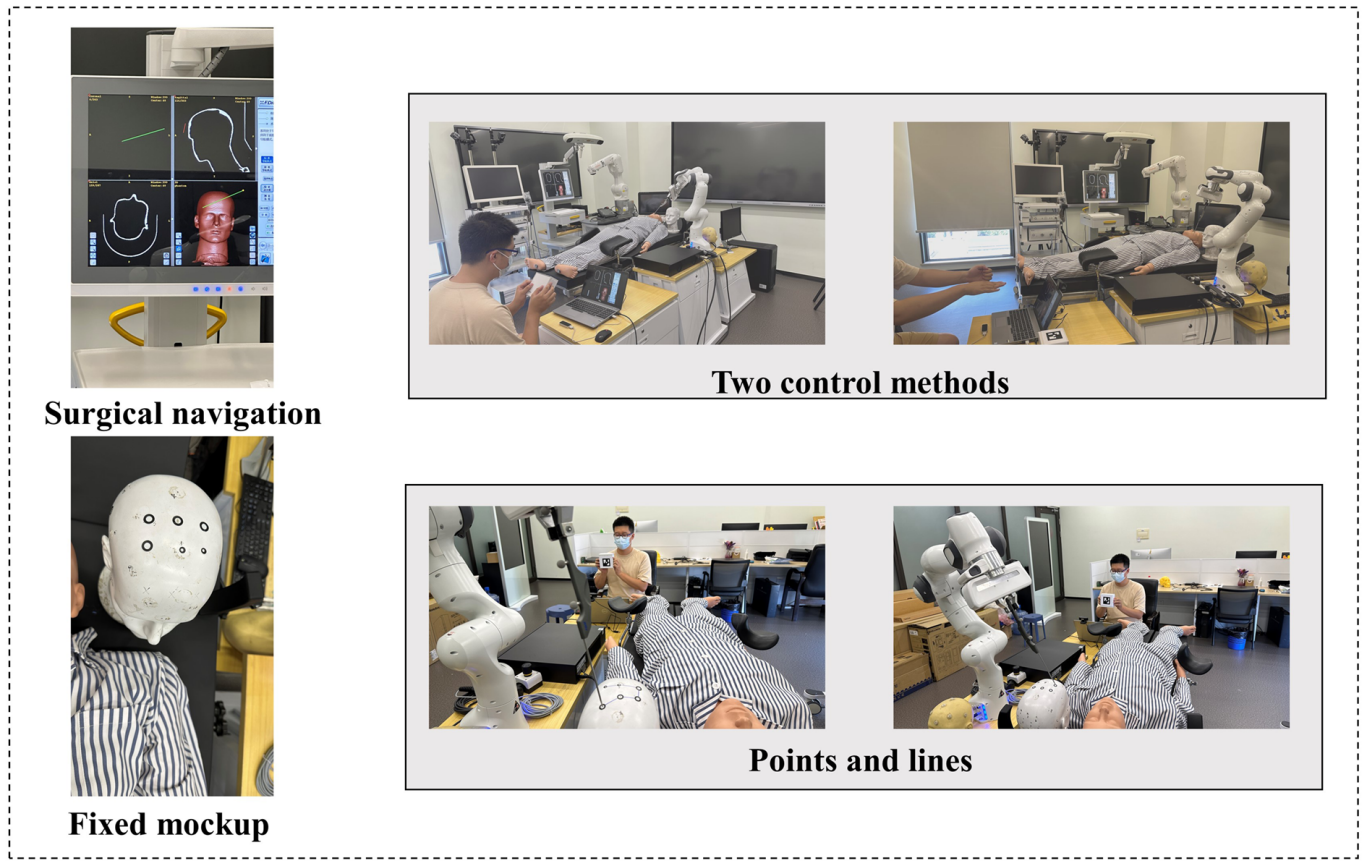
The flow of phantom experiment.

The interaction model is implemented on the collaborative robotic arm, Franka Emikia, and its effectiveness is verified through experiments, following the process outlined in Figure 8.

### 3.1. LeapMotion hand recognition accuracy

To integrate our gesture recognition module into a robot, we first conducted an accuracy test on our module. We recruited 10 volunteers and evaluated the success rate of six gestures on average. Each volunteer performed each gesture five times, resulting in a time range of 2 min 10 s to 3 min 15 s for the task to be completed. These findings demonstrate that our gesture-based operation method is not only user-friendly but also effective, even for beginners. The actions we designed are reasonable and efficient.

### 3.2. Aruco recognition accuracy

Different from gestures, as shown in Table 2, using Aruco to operate can represent 12 types of instructions for fine-tuning the position of surgical instruments. Specifically, the difference lies in two aspects. Firstly, there are six more rotation instructions than gesture operations. Secondly, the operation using Aruco will be much more precise. For fine-tuning after adjusting the instrument using hand gestures. We had 10 volunteers attempt to trigger 12 different commands, and their success rates are as follows. In addition, the same target experiment was carried out, but this time volunteers were allowed to use a combination of both modes of operation. The result was a significant reduction in time, with an average of 25 s shaved off. The main reason for the improved operating efficiency is the simplicity and convenience of the newly designed control method.

**Table 2:** Accuracy of Aruco recognition with predefined categories.

Category	1	2	3	4	5	6
Accuracy	97%	94%	95%	96%	97%	98%
Category	7	8	9	10	11	12
Accuracy	98%	99%	99%	96%	98%	98%

### 3.3. Phantom experiment

In this experimental part, under the guidance of the surgical navigation system, we used LeapMotion and Aruco tags to control the surgical robot to hold the puncture instrument and reach the preset point. And with the help of the surgical system we can calculate the error in position (Table 3).

**Table 3:** Error of the needle insertion in phantom experiment.

Number of experiments	Position error (mm)
1	1.25
2	1.20
3	1.06
4	1.00
5	1.15

#### 3.3.1. Experimental settings

In this experiment, the volunteers will move according to the predetermined route on the target on the skull model. In this process, the volunteers will first use gestures to control the surgical instruments to move faster, and then use Aruco to control them when they reach the target point. We analyzed the planned surgical path and the actual surgical path, and obtained the error of the path.

#### 3.3.2. Experimental result

The five experiments in Table 2 show the average alignment error of the needle tip, and the experimental data show that the average alignment error is 1.13 mm.

## 4. Discussion

In this research, we propose a novel pose recognition framework and integrate it into a robot, successfully completing related operations in various task sets. Compared to traditional manual operation, remote teleoperation based on human body pose estimation is highly feasible and can be seamlessly integrated into existing robots, enabling remote-operated robotics under various conditions. This is particularly evident in the medical task of puncture operation, where remote operation minimizes the risk of germ spread, reduces the time required for disinfection, and enhances the efficiency of surgery.

Although the current system design has shown good performance in various tasks, there are still some limitations to be addressed. In the trajectory tracking task, the robot did not move precisely along the set trajectory, resulting in slight trajectory fluctuations. This may have contributed to the poor network communication effect, causing the robot to receive the attitude estimation signal from the operator with a delay. As a result, the operator had to continuously adapt their posture to operate the robot, leading to an over-correction phenomenon. However, these limitations can be overcome by leveraging 5G technology and dedicated network lines. Additionally, in the robot-assisted puncture surgery task, although the robot completed the task flawlessly under remote operation by the operator, the operator lacked the tactile feedback and force feedback that are essential in a real surgical setting. As a result, the operator had to rely more heavily on the visual information provided by the navigation system throughout the procedure.

The current system design allows for remote teleoperation through the operator’s posture, with the assistance of relevant visual information. Experimental results demonstrate that the proposed system can effectively control surgical instruments for both large-scale movements and fine-tuning in a non-contact scenario.

## 5. Conclusion

In this work, a novel human-computer interaction method based on LeapMotion and Aruco is proposed and applied in contactless robotic surgery. This approach allows for a more hygienic and cost-effective surgical experience compared to traditional methods of grasping the robotic arm with the surgeon’s hand. By leveraging the guidance of surgical navigation systems, the position of surgical instruments can be accurately and quickly adjusted, streamlining the surgical process. Our proposed method has been proven effective and robust through previous experiments, and holds significant practical potential in clinical settings. Moving forward, the upgrade of sensors and optimization of algorithms can further expand the auxiliary functions of surgical robots, providing stronger support for the surgical system.

## Data availability statement

The original contributions presented in the study are included in the article/supplementary material, further inquiries can be directed to the corresponding authors.

## Author contributions

XZ: Writing – original draft, Methodology. JW: Methodology, Software, Writing – original draft. XD: Project administration, Software, Writing – original draft. SS: Writing – review & editing. XC: Funding acquisition, Writing – review & editing.
